# Abstract of Proceedings of the Buffalo Medical Association

**Published:** 1865-09

**Authors:** Joseph A. Peters

**Affiliations:** Secretary


### ART. II—*Abstract of Proceedings of the Buffalo Medical Association.*

TUESDAY EVENING, July 11, 1865.

The Association met pursuant to adjournment, the President, Dr. Ring, in the Chair. Present, Drs. Rochester, Congar, Strong, Gould, Samo, Boardman, Smith, Johnson and Peters.

The report of prevailing diseases was taken up, when

DR. ROCHESTER called attention to the prevalence of whooping cough, and to the fact that it was a much more dangerous disease in very young children than was generally supposed. He thought physicians should take pains to let it be known, in the families which they attended, as parents were very apt to consider it a disease of little moment. As to treatment, he had been inclined early in the epidemic to rely chiefly upon bromide of ammonium, but further experience had led him to place a less high estimate upon its efficacy. Not wishing to recommend a remedy too hastily, he felt bound to put on record the fact that it had often failed in his hands. In one case where the bromide of ammonium had failed, he had had very good success with the combination of sulphate of zinc and belladonna.

DR. BOARDMAN had seen several severe cases of urticaria.

Some discussion also took place concerning the treatment of whooping cough, by those present—the weight of evidence seeming to be in favor of the sulphate of zinc and belladonna.

Dr. Peters inquired whether the gentlemen present had seen in any cases, poisoning from opium take place without sleep being produced. Was induced to make this inquiry from having had occasion to note the effects of large doses of morphia. Wished also to call attention to the danger of administering this drug at the same time with chloroform, which he believed was often done with too little care. Referred especially to morphia because he believed it more dangerous than other preparations of opium.

Dr. Boardman had seen one case of a gentleman who had been accustomed to large doses of opium, but who took too much one afternoon, was attacked with severe and persistent vomiting which lasted all next day, death taking place in about twenty-four hours. Had no doubt death might result from opium without sleep having been induced.

Dr. Rochester said little children sometimes died from the effects of opium when there was no sleep until the last. In a certain class of cases opium often produces convulsions. Thought it often had an enfeebling and prostrating effect, and should be given, especially to children, with great care.

Dr. Strong had always considered opium to have a stimulant, instead of a prostrating effect. At two hours' intervals, in moderate doses, he had always got a stimulant effect. Believed it could be given in *some* dose to any child.

Dr. Rochester did not intend to say it should never be given to children, but only that it should be used carefully. Wished to be understood as saying it was often given too long, especially in cases of cholera infantum. It interfered with alimentation, and hence was weakening when given for a long time. Believed it was used too much by all physicians of the present day; and while he would not by any means undervalue it, he would urge care in its use. In regard to the use of both opiates and anæsthetics, would say that they could be used together, and formed valuable adjuncts to one another.

Dr. Smith related a case of convulsions following an attempted abortion, which had occurred in his practice, when he administered enormous doses both of laudanum and morphia without effect, until he administered chloroform, when the woman slept well.

Dr. Johnson related a somewhat similar case which he had seen. Considered the two remedies very valuable in such cases.

Dr. Peters was well aware that the effects of chloroform were prolonged by opium, and *vice versa*, hence the danger. Did not intend to object to their being used together, but desired to point out the danger of giving chloroform after large doses of opium had apparently failed.

The Association adjourned.

Tuesday Evening, August 1st, 1865.

Association met pursuant to adjournment, the President, Dr. Ring, in the Chair. Present, Drs. Rochester, Samo, Trowbridge, Greene, Gleason, Cronyn, Wetmore, Johnson and Peters.

Drs. J. N. Browne and E. E. Little were elected members.

Dr. Trowbridge presented, through the Secretary, a copy of the tables of height, measurements, etc., of men examined in the Provost Marshal's office, which he had been required to make to the Provost Marshal General.

On motion of Dr. Rochester the thanks of the Association were presented to Dr. Trowbridge for the document, and the Secretary was instructed to preserve it in a form convenient for reference. Its great size renders its publication impossible.

Reports on prevailing diseases being called for, Dr. Rochester reported cholera infantum, whooping cough, and cerebro-spinal meningitis, as prevailing, and typhoid fever as measurably frequent. Thought cerebro-spinal meningitis more frequent among females than males, and among children from 8 to 18 years of age, than adults.

Dr. Cronyn had not seen a single case of uncomplicated whooping cough. Reported several cases of cerebro-spinal meningitis.

Dr. Greene had seen a good deal of whooping cough, and had found a good remedy in the following formula:

℞ Ground Coffee, ℥iv.
Water, - O ss.

Make a strong decoction, to which add

Tr. Veratrum Viride, ʒij.

Fluid Extract of Conium, ʒiv.

Dr. Ring reported the prevalence of typhoid fever, cholera infantum, cholera morbus, etc. Had tried with good effect in whooping cough, bromide of ammonium in syrup of tolu.

The Association adjourned.

Note.—The Secretary inserts here an account of a curious and interesting case which was related by Dr. Whitney at the December meeting, but has been delayed by Dr. W.'s illness.

Dr. Whitney related a case of partial paralysis of the arm consequent upon caries and exposure of the nerve of the lower dens-sapientia of the right side. The patient was a thin, spare woman, about forty years of age, of decided nervous temperament—had had very little pain in the tooth, but for several weeks or months, considerable pain in the right side of the neck, extending to the shoulder and arm, with rigidity of the muscles, and, at times, immobility of the arm. On raising the hand to the face to locate the pain, it fell to her side. On coming in contact with the nerve in probing the tooth, the effect was more manifest in the arm than in the tooth, by painful twitching of the muscles, with an inability to raise it, so much so that she took hold of it with the other hand. He was now fully satisfied that the trouble in the arm, that had nearly deprived her of the use of the needle, and given her so much anxiety, and had been treated with fomentations, lotions, friction, etc., was referable to the condition of the tooth by *reflex* action. The usual mode of devitalizing and removing the pulp and filling the cavity entirely cured the other annoyances at once.

Joseph A. Peters, Secretary.

